# Alterations in rat prefrontal cortex kynurenic acid levels are involved in the enduring cognitive dysfunctions induced by tetrahydrocannabinol exposure during the adolescence

**DOI:** 10.3389/fpsyt.2022.996406

**Published:** 2022-11-22

**Authors:** Sarah Beggiato, Alessandro Ieraci, Mariachiara Zuccarini, Patrizia Di Iorio, Robert Schwarcz, Luca Ferraro

**Affiliations:** ^1^Department of Medical, Oral and Biotechnological Sciences, University of Chieti-Pescara, Chieti, Italy; ^2^Department of Life Sciences and Biotechnologies, University of Ferrara, Ferrara, Italy; ^3^Department of Pharmaceutical Sciences, University of Milan, Milan, Italy; ^4^Department of Theoretical and Applied Science, eCampus University, Novedrate, Italy; ^5^Department of Psychiatry, Maryland Psychiatric Research Center, University of Maryland School of Medicine, Baltimore, MD, United States; ^6^Laboratory for the Technology of Advanced Therapies (LTTA Centre), University of Ferrara, Ferrara, Italy

**Keywords:** schizophrenia, kynurenine pathway, KAT II inhibitor, glutamate, THC

## Abstract

**Introduction:**

Cannabis abuse during adolescence is a risk factor for cognitive impairments in psychiatric disorders later in life. To date, the possible causal relationship between cannabinoids, kynurenic acid (KYNA; i.e., a neuroactive metabolite of tryptophan degradation) and cognition has not been investigated in adolescence. Early exposure to delta 9-tetrahydrocannabinol (THC; i.e., the main psychotropic component of cannabis) causes enduring cognitive deficits, which critically involve impaired glutamatergic function in the prefrontal cortex (PFC). In addition, prenatal cannabis exposure results in enduring increases in PFC KYNA levels. Based on these findings, the effects of chronic THC exposure in rats, during another critical period of neurodevelopment particularly sensitive to perturbation by exogenous stimuli, such as adolescence, have been investigated.

**Methods:**

Male Wistar rats were chronically treated with vehicle or ascending intraperitoneal (i.p.) doses of THC starting on postnatal day (PND) 35 until PND 45. In adulthood (PND 75), cognitive assessment (Y-maze) and extracellular KYNA/glutamate levels were measured in the PFC by *in vivo* microdialysis, before and after a challenge with KYN (5 mg/kg i.p., the biological precursor of KYNA). By using the selective, brain-penetrable KAT II inhibitor PF-04859989, we then examined whether blockade of KYNA neosynthesis prevents the cognitive impairment.

**Results:**

Compared to vehicle-treated controls, extracellular basal KYNA levels were higher in the PFC of adult rats chronically exposed to THC in adolescence (*p* < 0.01). No changes were observed in extracellular glutamate levels. Following a challenge with KYN, extracellular KYNA levels similarly increased in both groups (i.e., vehicle- and THC-treated; *p* < 0.001 and *p* < 0.01, respectively). Chronic adolescent THC exposure negatively affected short-term memory (reduced spontaneous alternation), in adult animals (*p* < 0.001), while PF-04859989 (30 mg/kg i.p.) restored the cognitive impairment (*p* < 0.05).

**Discussion:**

We propose that the observed alterations in PFC KYNA signaling might be involved in the cognitive dysfunction induced by the exposure to THC during the adolescence. In the translational realm, these experiments raise the prospect of prevention of KYNA neosynthesis as a possible novel approach to counteract some of the detrimental long-term effects of adolescence cannabis use.

## Introduction

Over the recent past years, changes in the legal status of recreational or/and medical cannabis use have led to increased utilization of the drug among adolescents in the USA and Europe (1, 2). In addition, preliminary data indicate that the frequency of cannabis (as well as alcohol) use has increased during the COVID-19 pandemic (3). Since cannabis availability continues to expand, there is now growing concern about the possible negative effects of the main psychotomimetic component of cannabis, delta 9-tetrahydrocannabinol (THC), particularly during adolescence and other sensitive periods of brain development (4). Specifically, prolonged and heavy use of cannabis is a risk factor for cognitive impairments in psychiatric disorders later in life, especially in subsets of vulnerable adolescents (5).

During adolescence, the brain is characterized by increased plasticity and neuronal circuits refinement, specifically in the prefrontal cortex (PFC), one of the last brain regions to reach full development, and a brain area contributing to behavioral, cognitive and emotional maturity (6, 7). In addition, nowadays cannabis variants contain increasingly higher concentration of THC, multiplying the potency of the psychoactive ingredient and thereby increasing negative effects on several brain areas including the PFC (8). Cannabis abuse in adolescents is linked to cognitive impairments with a greater susceptibility to long-lasting consequences in teens who begin using cannabis at an earlier stage. Specifically, these cognitive impairments are related to the domains of attention, processing speed, verbal learning and memory, and executive functioning (9–11).

Studies in non-human primates have also reported a selective effect of THC for spatial working memory in adolescent monkeys following repeated drug administration (12). Preclinical studies in rodents indicate that adolescent cannabis exposure is associated with long-lasting changes in the structure of the cortical neurons in rats (13, 14) along with cognitive dysfunctions in adulthood (15, 16). Specifically, early exposure to THC in rats, causes enduring cognitive deficits, which critically involve impaired glutamatergic function in the PFC (6, 17).

**FIGURE 1:**
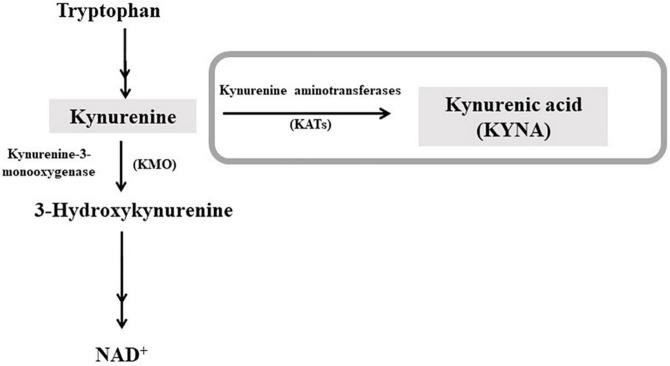
Schematic representation of the kynurenine pathway of tryptophan degradation. Kynurenic acid (KYNA) is synthesized from kynurenine by kynurenine aminotransferases (KATs). In the second ramification of the kynurenine pathway, kynurenine is metabolized by kynurenine 3-monooxygenase (KMO), leading to NAD^+^ as final product. For a complete representation of the pathway, see Schwarcz et al. (22).

Interestingly, it has been recently demonstrated the possible role of kynurenic acid (KYNA), an astrocyte-derived neuroactive metabolite of the kynurenine pathway (KP; Figure 1), in the long-term effects of cannabis exposure during brain development (18). Furthermore, the cognitive deficits induced by experimental increases in brain KYNA levels in developing rodents (19–21) are qualitatively very similar to those caused by cannabinoids. KYNA can function as an endogenous antagonist of α7-nicotinic acetylcholine receptors (α7nAChR) and N-methyl-D-aspartate receptors (NMDAR; (22), for review). KYNA is mainly synthesized from its biological precursor kynurenine (KYN), by astrocytic kynurenine aminotransferases (KATs), with KAT-II preferentially responsible for KYNA neosynthesis in mammals (23).

KYNA serves as a functionally significant neuromodulator in the mammalian brain, including the PFC. For example, even modest increases in cerebral KYNA levels induce bi-directional effects on neurotransmitter levels in brain regions of different rodent models. Furthermore, altered brain (including PFC) KYNA levels have been associated with several pathological conditions, as extensively described by Schwarcz et al. (22).

Increased KYNA levels are associated with deficits in cognitive functions, included working memory, sensorimotor gating and attentional processing impairments, in adult rats (19, 24–29). Accordingly, several clinical studies report a pathophysiologically relevant association between increased brain KYNA levels and cognitive dysfunction in individuals with schizophrenia (SZ). In addition, several authors reported that SZ people have increased KYNA levels in the PFC (30, 31) and cerebrospinal fluid (32, 33). This elevation in KYNA levels can also lead to neurotransmission changes, as fluctuations in KYNA normally control aminoacidergic, cholinergic and dopaminergic neurotransmission in the mature brain (34, 35), while the specific inhibition of KYNA synthesis by KAT II inhibitors, improves such cognitive deficits [(22), for review; (21, 36)].

Based on the previous findings demonstrating that the exposure to THC during the prenatal period significantly biased KYNA in the rat PFC at adulthood [*see* above; (18)], the aim of the present study was to explore the possible consequences, on KP metabolism, of cannabinoid consumption in another critical period of brain development, particularly sensitive to perturbation by exogenous stimuli, i.e., the adolescence. To date, the possible causal relationship between cannabinoids consumption and brain KYNA levels, along with their possible impact on cognitive functions have not been investigated in adolescence.

## Materials and methods

### Chemicals

Delta 9-tetrahydrocannabinol (THC), L-kynurenine sulfate (KYN) and PF-04859989 were purchased from Sigma (Milan, Italy). THC was dissolved in ethanol, cremophor and saline (1:1:18). This solution has been administered to vehicle-treated (i.e., control) rats. KYN and PF-04859989 were dissolved in saline solution.

### Animals and exposure conditions

#### Animals

Male Wistar rats aged 28 days at the time of arrival were obtained from Charles River laboratories (Calco, Italy) and were housed in clear plastic cages on a 12 h light-dark cycle (lights on 08:00 h) and in a temperature (22 ± 2°C) and humidity controlled environment (50 ± 10%). All animals were allowed free access to food and water. All experiments took place during the light phase.

The study was carried out in accordance with current Italian legislation (D.L. 26/2014) that allows experimentation on laboratory animals only after the approval by Ministry of Health (Rome, Italy), and in strict accordance with the European Council Directives on animal use in research (n. 2010/63/EU). Accordingly to the ARRIVE guidelines, all possible efforts were made to minimize animal pain and discomfort and to reduce the number of experimental subjects. 46 rats have been used in this study.

#### Adolescent tetrahydrocannabinol treatment

**FIGURE 2:**
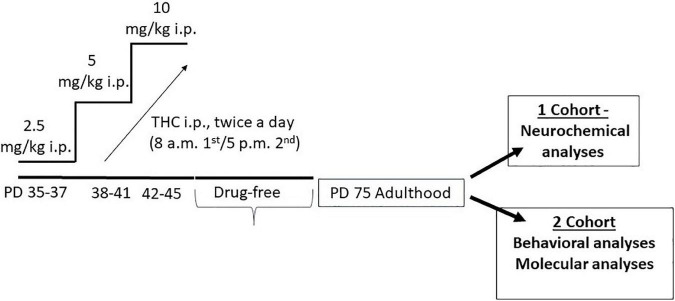
Schematic representation of adolescent tetrahydrocannabinol treatment protocol and experiment timeline.

Male rats were housed in pairs, one rat receiving THC and the other one receiving vehicle treatment. Specifically, the animals were injected with increasing doses of THC, or vehicle, twice a day from postnatal day (PND) 35 to PND 45 (2.5 mg/kg, PND 35–37; 5 mg/kg, PND 38–41; 10 mg/kg, PND 42–45), according to our previous published protocol. This protocol mimics a heavy use of marijuana, since, according to the transformation in human equivalent dose proposed by FDA and the average content of THC in a joint, our first dose roughly corresponds to one joint, the second one to two joints, and the higher one to four joints (17). These moderate to high doses were chosen to be within the range known to produce behavioral effects in rats. Animals were then left undisturbed till adulthood. At PND 75 a first cohort of adolescent THC- and vehicle-treated rats was implanted with a microdialysis probe for neurochemical experiments, while a second cohort was used for behavioral and molecular studies (Figure 2).

## Neurochemical analyses

### Microdialysis experiments

#### Surgery

On the day of microdialysis probe implantation (PND 75), the animals were kept under isoflurane anesthesia (1.5% mixture of isoflurane and air) and mounted in a David Kopf stereotaxic frame (Tujunga, CA, USA) with the upper incisor bar set at 2.5 mm below the interaural line. A small hole was drilled on one side of the exposed skull. A microdialysis probe of concentric design (CMA12; molecular weight cutoff: 20 kD; outer diameter: 0.5 mm; length of the dialysis membrane: 2 mm; Alfatech S.p.A., Genova, Italy) was implanted vertically in the medial PFC (mPFC) and then secured to the skull with anchor screws and acrylic dental cement. The coordinates used [expressed in millimeters from bregma, according to Paxinos and Watson’s atlas Paxinos and Watson (37)] were: anterior 3.5, lateral 0.8 and vertical – 4.0 for adult rats. After surgery, the animals were housed individually in microdialysis chambers with food and water available.

#### Microdialysis

Experiments were performed in freely moving rats 24 h after the probe implant. The probe was connected to a microperfusion pump (CMA 100; Carnegie Medicin, Stockholm, Sweden) set to a speed of 1.5 μl/min and perfused with Ringer solution containing (in mM): NaCl, 144; KCl, 4.8; MgSO4, 1.2; CaCl2, 1.7; pH 6.7. The collection of dialysate samples commenced 300 min after the onset of perfusion to achieve stable dialysis glutamate levels (18, 38) and perfusates were collected every 30 min thereafter. Following the collection of three stable basal values, KYN (5 mg/kg) or saline was administered i.p. and other nine perfusate samples were then collected (total sample collection time: 360 min).

**FIGURE 3:**
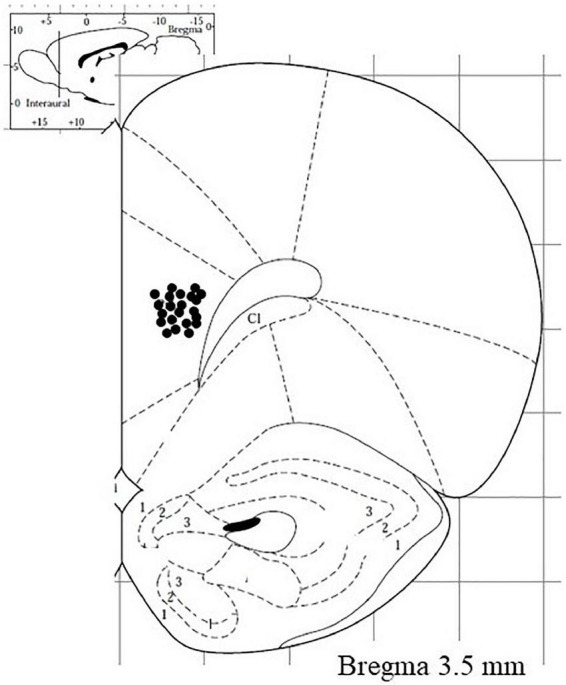
Visual representation of microdialysis probe tracks (deeper point) in the medial prefrontal cortex of rats subjected to neurochemical experiments.

Following each experiment, the brain was removed from the skull, and the position of the dialysis probe was verified using 30 μm-thick coronal cryostat sections. Only those animals in which the probe was correctly located were included in this study (Figure 3).

#### Kynurenic acid analysis

The extracellular concentration of KYNA in microdialysates was measured by HPLC (18). Twenty μl of the dialysate were injected onto a 3 μm C18 reverse phase HPLC column (100 mm × 4 mm; Dr. Maisch GmbH, Ammerbuch, Germany), using a mobile phase containing 50 mM sodium acetate and 4% acetonitrile (pH adjusted to 6.2 with glacial acetic acid) at a flow rate of 0.5 ml/min. Zinc acetate (0.5 M, not pH adjusted) was delivered post-column by an auxiliary pump (Dionex AXP, Thermo Fisher, Waltham, MA, USA) at a flow rate of 0.1 ml/min. In the eluate, KYNA was detected fluorimetrically (excitation: 344 nm, emission: 398 nm; Jasco fluorescence spectrophotometer FP2020 Plus, Jasco, Tokyo, Japan). The retention time of KYNA was approximately 14 min. The limit of detection for KYNA was 10 fmol/sample.

#### Glutamate analysis

In the dialysate, glutamate was measured by HPLC with fluorimetric detection. Briefly, 10 μl were transferred into glass microvials and placed in a temperature-controlled (4°C) Triathlon autosampler (Spark Holland, Emmen, Netherlands). Fifteen μl of o-phthaldialdehyde/mercaptoethanol reagent were added to each sample, and 15 μl of the mixture were injected onto a Chromsep analytical column (3 mm inner diameter, 10 cm length; Chrompack, Middelburg, Netherlands). The column was eluted at a flow rate of 0.48 ml/min (Beckman 125 pump; Beckman Instruments, Fullerton, CA, USA) with a mobile phase containing 0.1 M sodium acetate, 10% methanol and 2.2% tetrahydrofuran (pH 6.5). Glutamate was detected by means of a Jasco fluorescence spectrophotometer FP2020 Plus (Jasco, Tokyo, Japan). The retention times of glutamate was approximately 3.5 min. The limit of detection for glutamate was 20 fmol/sample (18).

### Behavioral analyses

#### Y-maze

The effect of chronic adolescent THC exposure on short-term memory was evaluated by using the Y-maze test. Furthermore, based on the obtained results, the effect of an acute administration of the KAT II inhibitor (PF-04859989; 30 mg/kg i.p., 1 h prior to behavioral testing) has also been tested. Namely, the following 4 groups of rats have been compared: (1) adolescent vehicle-treated rats receiving saline; (2) adolescent THC-treated rats receiving saline; (3) adolescent vehicle-treated rats receiving PF-04859989 and (4) adolescent THC-treated rats receiving PF-04859989.

The maze consisted of three interconnected closed arms, each one of them is 120° from the adjacent. Individual rat was placed into the middle of the maze and was allowed to freely explore the maze for 5 min. The number and order of arm entries were recorded and analyzed using the Any-maze video tracking system (Ugo Basile, Varese, Italy). An alternation was determined as the number of consecutive entries into the three different arms. The number of arm entries was used as a marker of locomotor activity. The number of spontaneous alternations (i.e., the entry into three different arms in sequence (triad), was used as a measure of short-term working memory. The percentage of spontaneous alternations was calculated from the number of triads and arm entries using the following equation: *Y* = number of triads/(total number of arm entries-2) × 100 (39, 40).

On the day after the behavioral test (PND 76), the animals were sacrificed by decapitation under isoflurane anesthesia. Animals were euthanized using an anesthetic overdose of isoflurane. The brains were rapidly removed and both the PFC dissected out frozen on dry ice and stored at –80°C until the day of the molecular analysis.

### Molecular analyses

#### Ribonucleic acid isolation and reverse transcription

Ribonucleic acid (RNA) isolation and reverse transcription was performed as previously described (18). Briefly, total RNA from the PFC was extracted using the Direct-zolTM RNA MiniPrep (Zymo Research, purchased by Euroclone, Milan, Italy) according to manufacturer’s instructions. cDNA was synthesized using the iScript kit (Biorad, Milan, Italy) according to manufacturer’s instructions.

#### Quantitative real-time PCR

Quantitative real-time PCR (qPCR) analysis was performed on a CFX Connect Real-Time System (Biorad) using the iTaq Universal SYBR Green supermix (Biorad) and the following primers: KAT I Fw: 5′-CAATGATGGCTGGAGGTTG-3′, Rev: 5′-GTTGTTGGGTGTGTTGAGGA-3′; KAT II Fw: 5′-CCCTG TACTTTATCACAGCTC-3′, Rev: 5′ AAACCACGTAACCACT TGTC-3′; KAT III Fw: 5′-TTCAAAAATGCCAAACGAATCG-3′, Rev: 5′-GATGACCAAAGCCCCTTGTGT-3′; KAT IV Fw: 5′-GGACCTCCAGATCCCATCCT-3′, Rev: 5′-GGTTTTCCG TTATCATCCCGGTA-3′; S18: Fw: 5′-CATGCAGAACCCAC GACAAT-3′, Rev.: 5′-CTTCCCATCCTTCACGTCCT-3′. qPCR conditions were: 10 min at 95°C, 40 cycles of 15 s at 95°C and 30 s at 60°C. Relative expression of mRNA was calculated with the comparative Ct (ΔΔCT) method using RPS18 as endogenous control gene. The relative mRNA levels were expressed as fold change. Melting curve analysis was performed to verify the specificity of the PCR products (40).

### Data management and statistical analysis

*In vivo* microdialysis data were not adjusted for recovery from the dialysis probe. Basal values were calculated as the mean of three consecutive samples (differing by no more than 15%) collected immediately preceding the drug treatment. The area created by the curve (AUC), reflecting the duration of the effect, was determined for each animal by using. Area values (overall effects) were calculated as percentages of changes in baseline value over time by using the trapezoidal rule.

Data are expressed as the mean and SEM. For microdialysis experiment, Student’s *t*-test was used to analyze the differences in the mean KYNA and glutamate basal levels, while the effects of KYN injection were statistically analyzed by three-way (time-course) or two-way (AUC) analysis of variance (ANOVA) followed by Tukey’s test for multiple comparisons when appropriate. In the Y maze test statistical differences were assessed by two-way ANOVA with THC exposure and PF-04859989 (KAT II inhibitor) as main factors, followed by Tukey’s test for multiple comparisons. For molecular analyses, data were analyzed by Student’s *t*-test.

## Results

### Neurochemical data

#### Effect of adolescent THC exposure on basal extracellular kynurenic acid and glutamate levels in the medial PFC of adult male rats

**FIGURE 4:**
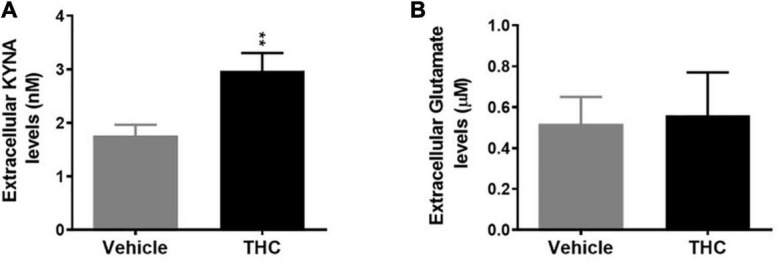
Basal extracellular kynurenic acid (KYNA; panel **A**) and glutamate (panel **B**) levels in the medial prefrontal cortex of adult [post-natal day (PND) 75] male rats chronically exposed to delta 9-tetrahydrocannabinol (THC) or its vehicle (cremophor and saline 1:1:18; Vehicle) during the adolescent period. Ascending doses of THC or its vehicle were administered by i.p. to adolescent rats, from PD 35 to PD 45, wh. Data are the mean ± standard error of the mean (SEM); *n* = 5/6 male rats/group. **p < 0.01 significantly different from the respective vehicle group based on Student’s *t*-test.

*In vivo* microdialysis experiments indicate that chronic adolescent THC exposure differently affected KYNA and glutamate levels in the mPFC of adult rats. In fact, extracellular KYNA levels were significantly increased (Figure 4A, *p* < 0.01), while extracellular glutamate levels were not affected (Figure 4B), in adult animals exposed to THC during the adolescence, as compared to vehicle-treated animals.

#### Effect of a pharmacological challenge with kynurenine on extracellular kynurenic acid and glutamate levels in the medial PFC of adult male rats chronically exposed to THC during adolescence

To further investigate the impact of chronic adolescent-THC treatment on the KP, we evaluated the consequences of an acute challenge with KYN (5 mg/kg, i.p.), the direct bioprecursor of KYNA, on extracellular KYNA and glutamate levels in the mPFC of adult male rats.

**FIGURE 5:**
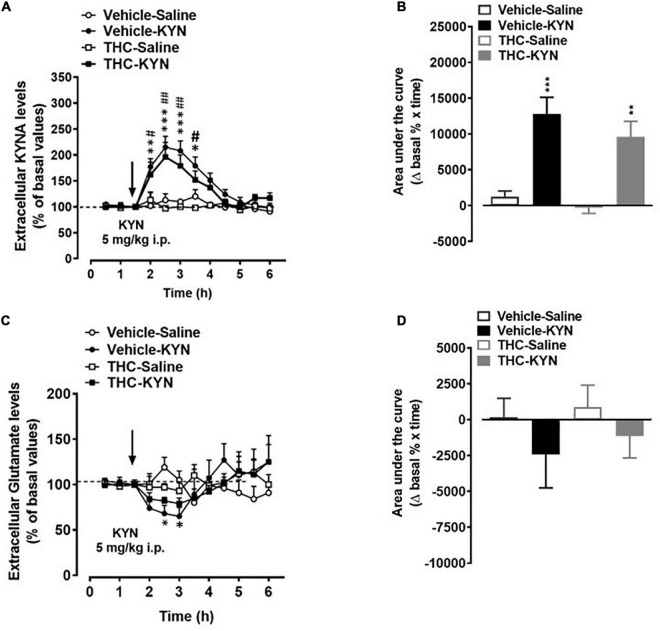
Extracellular kynurenic acid (KYNA; panel **A,B**) and glutamate (panel **C,D**) levels in the medial prefrontal cortex of adult [post-natal day (PND) 75] male rats chronically exposed to delta 9-tetrahydrocannabinol (THC) or its vehicle (cremophor and saline 1:1:18; vehicle). Ascending doses of THC or its vehicle were administered by i.p. to adolescent rats, from PD 35 to PD 45. **(A,C)** Data mean ± standard error of the mean (SEM) are expressed as a percentage of the averaged three baseline values prior to treatment. **(B,D)** Bar graphs of the areas under the curves (AUC), calculated as the percentage of changes in basal values over time. Data were analyzed by three-way **(A,C)** or two-way **(B,D)** ANOVA and subsequent Tukey’s *post hoc* test for multiple comparisons. **(A)** *p < 0.05, **p < 0.01, ***p < 0.001 Vehicle KYN *vs* Vehicle-Saline and THC-Saline; ^#^*p* < 0.05, ^##^*p* < 0.01 THC-KYN *vs* Vehicle-Saline and THC-Salina; **(B)** ***p < 0.001 Vehicle-KYN vs Vehicle-Saline and THC-Saline; **p < 0.01 THC-KYN vs Vehicle-Saline and THC-Saline. **(C)** *p < 0.05 Vehicle-Saline vs Vehicle-KYN and THC-Saline; *n* = 5/6 male rats/group.

In vehicle and THC groups, KYN administration induced a similar increase in extracellular KYNA levels, as assessed by evaluating the nadir of the effect (Figure 5A) or the area under the curve (AUC) results (Figure 5B). In fact, a three-way ANOVA (time-course; Figure 5A) showed significant effects of KYN (*F*_(1,5)_ = 46.40, *p* = 0.0010), time (*F*_(11,55)_ = 30,37, *p* = 0.0001) and time × KYN (*F*_(11,55)_ = 20.71, *p* = 0.0001), but non-significant effects of THC, THC × KYN and THC × KYN × time. The *post hoc* test showed similar significant increases in KYNA levels 30, 60, 90 and 120 min after KYN injection in vehicle and THC groups. Two-way ANOVA (AUC; Figure 5B) showed significant effects of KYN (*F*_(1,19)_ = 40.93, *p* = 0.0001), but non-significant effects of THC and THC × KYN. On the other hand, KYN challenge induced a slight reduction of extracellular glutamate levels in the mPFC of vehicle-treated (Figure 5C). Three-way ANOVA (time-course; Figure 5C) showed significant effects of time (*F*_(11,55)_ = 3.333, *p* = 0.0014) and time × KYN (*F*_(11,55)_ = 11.00, *p* < 0.0001), but non-significant effects of THC, KYN, THC × KYN, time × THC and time × THC × KYN). The *post hoc* test showed a significant decrease in glutamate levels 60 and 90 min after KYN injection in the vehicle group. However, the overall AUC results did not show any statistically difference among the groups. In fact, two-way ANOVA (AUC; Figure 5D) showed non-significant effects of THC, KYN and THC × KYN.

### Behavioral data

#### Effect of chronic adolescent THC exposure on short-term memory performance in adult male offspring

**FIGURE 6:**
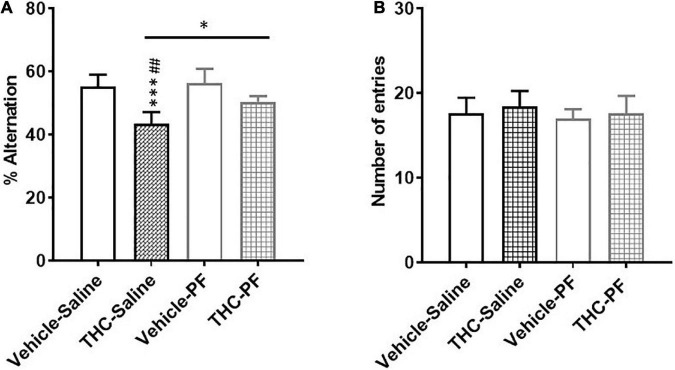
Y-maze test showing the percentage of spontaneous alternations **(A)** and the number of arm entries **(B)** in adult rats [post-natal day (PND) 75] chronically exposed to delta 9-tetrahydrocannabinol (THC) or its vehicle (cremophor and saline 1:1:18; Vehicle) during the adolescent period. Ascending doses of THC or its vehicle were administered by i.p. to adolescent rats, from PD 35 to PD 45. Bars represent the mean ± standard error of the mean (SEM; *n* = 5/6 male rats/group). Data were analyzed by two-way ANOVA and subsequent Tukey’s *post hoc* test for multiple comparisons. ^***^p < 0.0001 THC-Saline vs Vehicle-Saline; ^##^*p* < 0.01 THC-Saline vs Vehicle-PF; *p < 0.05.

The effect of chronic adolescent THC exposure on short-term memory was evaluated by using the Y-maze test. Furthermore, based on the obtained results, the effect of an acute administration of the KAT II inhibitor (PF-04859989; 30 mg/kg i.p.; 1 h prior to behavioral testing) on adolescent THC-induced cognitive impairment has also been tested. Overall, we observed main effects of chronic THC exposure (*F*_(1,19)_ = 34.31, *p* < 0.0001) and PF-04859989 treatment (*F*_(1,19)_ = 7.34, *p* < 0.013), and no interaction “chronic THC exposure” × “PF-04859989 treatment” (*F*_(1,19)_ = 3.69, *p* = 0.056). As shown in Figure 6, chronic adolescent THC exposure negatively affected (*p* < 0.0001) short-term memory (reduced spontaneous alternation, Figure 6A), but not locomotor activity (arm entries, Figure 6B), compared to the vehicle-exposed rats. The injection of PF-04859989 in adult rats chronically exposed to THC during adolescence, significantly (*p* < 0.05) ameliorated the cognitive impairment (Figure 6A), without affecting locomotor activity (Figure 6B). The KAT II inhibitor did not affect cognitive performance or locomotor activity in adult rats chronically exposed to vehicle during adolescence.

### Molecular data

#### Effects of chronic adolescent THC exposure on kynurenine aminotransferases enzymes expression in the medial PFC of adult male

**FIGURE 7:**
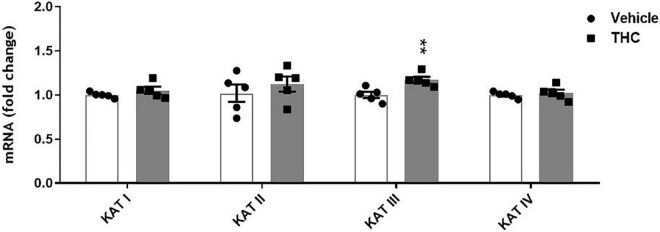
Enzyme expression of kynurenine aminotransferases (KATs) isoforms in prefrontal cortex tissues from adult [post-natal day (PND) 75] rats chronically exposed to delta 9-tetrahydrocannabinol (THC) or its vehicle (cremophor and saline 1:1:18; Vehicle) during the adolescent period. Ascending doses of THC or its vehicle were administered by i.p. to adolescent rats, from PD 35 to PD 45. Bars represent the mean ± standard error of the mean (SEM; *n* = 5/group). Data were analyzed by Student’s *t*-test. **p < 0.01 THC vs Vehicle.

We also evaluated the impact of chronic adolescent THC exposure on the expression of the enzymes directly linked to KYNA formation, such as the 4 isoforms of KATs (KAT I, II, III and IV). Unexpectedly, mRNA expression levels of KAT III were significantly elevated in tissues of PFC from adult prenatally THC-exposed rats compared to the vehicle-treated animals (*p* < 0.01, Figure 7), while no significant differences were observed in the mRNA expression levels of KAT I, II and IV.

## Discussion

In the present study we demonstrate for the first time that adolescent THC exposure in rats induces a persistent elevation of mPFC KYNA levels in adulthood. In line with previously published data [(17); see (14), for review], we observed that the adult rats exposed to THC during the adolescence display short-term memory deficits. Furthermore, in contrast to control animals, adult rats which had been treated with THC during adolescence showed a loss of KYNA-induced modulation of PFC glutamate levels when were challenged acutely with a systemic injection of a low dose of KYNA’s immediate bioprecursor KYN. Notably, an acute injection of the specific KAT II inhibitor PF-04859989 reversed the cognitive impairment in THC-exposed rats. Jointly, these results not only further support the relevance of functional links between THC and KYNA, but also suggest a potential new and translationally significant opportunities for therapeutic interventions.

Several lines of evidence support the hypothesis that abnormally elevated brain levels of KYNA, play a pathophysiologically significant role in SZ and other major neurodevelopmental disorders (41, 42). Preclinical studies in rodents suggest that KP impairments in these diseases may originate early in critical periods of neurodevelopment. It has been reported that exposure to KYN during the adolescent period, induced distinct cognitive impairments on spatial, contextual learning and memory in adulthood (19).

**FIGURE 8:**
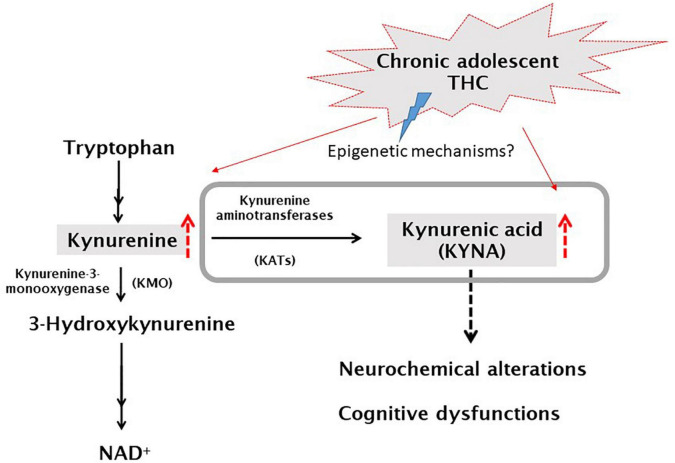
Simplified graphic representation of the kynurenine pathway and the current working hypothesis. Grey box represents what has been analyzed in this study. Hypothesized mechanism: ascending doses of delta 9-tetrahydrocannabinol (THC) administered twice a day (for details see section “Materials and methods”), to adolescent rats (PD35-45), would induce, possibly by epigenetic mechanisms, an increase of KYNA levels which, in turn, leads to neurochemical and cognitive dysfunctions [(22), for review].

Among the several hypotheses on SZ pathophysiology, also a dysfunction of the endocannabinoid system has been postulated (43, 44). KYNA shares multiple physiological functions with the endocannabinoid system in the central nervous system (CNS), including glutamate, acetylcholine, dopamine release regulation and control of learning and memory mechanisms (22, 45). This led to the assumption that the two systems can potentially interact with each other in the CNS, with potential future repercussions for therapeutic interventions. So far, there are limited data describing their actual cross-talk in the CNS, which clearly exist in the adult brain (46–49) although its precise relevance has yet to be ascertained. In a recent study we demonstrated that gestational exposure to THC causes, in rats, long-lasting increases of KYNA levels in the mPFC associated with cognitive deficits in adulthood (18). In the present study, we also demonstrate that cannabinoid consumption in another critical period of brain development, particularly sensitive to perturbation by exogenous stimuli (i.e., adolescence), induces long-term consequences on KP metabolism. Specifically, following *in vivo* microdialysis experiments, we observed, a significant increase of extracellular KYNA levels in the mPFC of adult male rats exposed to escalating doses of THC in adolescence. On the contrary, and in line with our previous study in female rodents, this treatment did not affect basal extracellular glutamate levels in male rats (17). Based on the well-recognized role of KYNA in reducing mPFC glutamate levels (50–52), the increase in KYNA levels observed in adult rats exposed to THC during the adolescence should be associated with a reduction in mPFC glutamate levels. Accordingly, we recently demonstrated that prenatal THC-induced long-term increase in mPFC KYNA levels was associated with a significant reduction in local glutamate levels (18). These observations suggest in the present study a lack of the tonic control induced by KYNA on mPFC extracellular glutamate levels in adolescent THC-exposed rats. This scenario is also supported by the evidence that, in adolescent THC-exposed rats, the impact of KYN administration on glutamate levels appears lower than that observed in the vehicle treated group (see below). However, due to the very slight effect of KYN on glutamate extracellular levels, this hypothesis remains to be confirmed in further experiments by using higher doses of KYNA precursor. Although the cellular and molecular mechanisms remain to be elucidated, it seems likely that epigenetic processes might be involved in the observed long-term effects of adolescence THC exposure (see Figure 8 for working hypothesis). Epigenetic factors are well-recognized as the principal mechanisms underlying enduring phenotypic changes, mainly due to the evidence that the epigenome provides the cellular context for environmental impacts, including cannabinoid exposure, to modify gene functions and associated behaviors (53). The enduring KYNA increase following adolescent THC treatment, may be associated with astrocyte activation. In fact, KYNA conversion takes places primarily in the astrocytes and previous studies reported, following chronic THC exposure during the adolescence, microglia and astrocyte activation associated with a persistent neuroinflammatory state specifically localized within the adult PFC (54, 55). This hypothesis is supported by the increase in KYNA levels induced by the addition of interleukin 6 to cultured human astrocytes, and the increased astrocytic activity observed in SZ individuals (42). Furthermore, some studies demonstrated that KATs expression/activity is increased under different inflammatory conditions (56–59). Although there are no data linking inflammatory state to the expression/activity of the different isoforms of KATs, it could be speculated that the hypothesized inflammatory state may also explain the small but significant increased expression of KAT III, which does not normally make significant contribution to KYNA neosynthesis in the mammalian brain (23, 60). However, it cannot be ruled out that the other KATs isoforms might be affected in their activity, and not expression, by adolescent THC exposure.

It has also been reported that THC exposure during adolescence alters dynamic changes occurring in glutamatergic and GABAergic signaling and desynchronization of PFC neuronal networks (55). Together with epigenetic modifications strongly affecting synaptic plasticity, these deleterious effects possibly explain the lack of the tonic control induced by KYNA on mPFC extracellular glutamate levels in adolescence THC-exposed rats. Epigenetic differences on specific global chromatin alterations within adult PFC and associated with glutamatergic and GABAergic systems following the same THC protocol in adolescent rats, have been already reported (61). Notably, previous studies, also reported a significant THC-induced attenuation of PFC GABAergic neurotransmission (16, 17, 62). Based on the present results, it can be postulated that the observed increased mPFC KYNA levels, could also contribute to adolescent THC-induced reduction in GABA signaling, since KYNA significantly reduces extracellular GABA levels in the rat PFC (52). This mechanism might be involved in adolescent THC-induced long-lasting cognitive impairment (see below), since increased KYNA levels has been associated with cognitive deficits (63) and an optimal interplay between inhibitory and excitatory neurotransmission is crucial in determining the functional integrity of adult PFC functioning (64–66). In this context, it becomes relevant to evaluate, in further studies, the possible impact of adolescent THC exposure on the PFC inhibitory/excitatory neurotransmission balance.

It is well-known that the negative effects of marijuana during adolescence might be due to disruption of the physiological role of the endocannabinoid system in this brain (including PFC) developmental window (67–69). According to the concept of higher vulnerability of the immature brain to external stimuli, it has been proposed that the exposure to THC during the adolescence may represent a risk factor for the development of a complex psychotic-like behavior in adulthood (16, 17). As previously postulated (18) this putative link is particularly captivating in persons with SZ, displaying elevated KYNA levels in brain and cerebrospinal fluid [see above; (30–33, 63)]. Based on the “two hit” hypothesis of SZ and considering adolescent THC exposure a “first hit,” in a second set of experiments we injected a low dose of KYN (5 mg/kg i.p.), the direct bioprecursor of KYNA to mimic a relevant “second hit.” The THC-exposed animals challenged with KYN, reported a similar quantitative increase on the extracellular KYNA levels as the vehicle exposed animals. This result diverges from our previous findings in prenatally THC exposed animals (18), displaying, when compared to vehicle-treated rats, a significantly greater increase in extracellular KYNA levels in the PFC following KYN injection. This difference might be obviously related to the different developmental period (i.e., prenatal or adolescence) of THC exposure and/or to the different doses of THC used in the present and in the previous study. Interestingly, the expected significant decrease in the extracellular glutamate levels associated to the increased KYNA levels (50, 52) was observed in vehicle-treated, but not in THC-exposed animals. This finding confirms the postulated (see above) lack of KYNA-mediated control on glutamate because of moderate to heavy escalating THC treatment performed in adolescence.

Based on the data obtained we hypothesize that the prolonged increase in PFC KYNA levels may be responsible for the cognitive impairment observed in the THC-exposed group. Since the spontaneous alternation in Y-maze test is strongly controlled by the PFC (70), these findings agree with the hypothesis that a dysfunctional PFC is associated with impairment of the executive functions (71). It has been widely reported that abnormal KYNA elevation is associated with cognitive deficits in pathological conditions like SZ (63). To date, the possible causal relationship between THC, KYNA and cognitive function has not been examined. In a first attempt to investigate this link, we determined the effect of an acute administration of the selective, brain penetrable KAT II inhibitor PF-04859989 (36). This approach has been used to test whether PF-04859989, by blocking KYNA neosynthesis and thus preventing the THC-induced delayed-KYNA increase, would ameliorate the associated impairments observed in the spontaneous alternation task. Administration of PF-04859989 rescued the cognitive impairment observed in the THC-exposed rats. Notably, preliminary *in vivo* data, also demonstrate that the single dose of the KAT II inhibitor was also associated with a greater reduction (∼40%) of extracellular KYNA levels in THC-exposed rats compared to the vehicle-rats (∼25%).

Although the molecular mechanisms, the role of brain circuits and the clinical relevance need to be still elucidated, the present finding has potential translational relevance and is in line with the fact that a pharmacologic reduction of cortical KYNA levels (36, 72) has evident pro-cognitive effects in several animal models. Thus, inhibition of KYNA neosynthesis by specifically targeting KAT II would constitute a potential new strategy to, at least partially, reduce cannabis-related adverse effects later in life.

Notably, it has been extensively shown that selective genetic or pharmacological interference with KYNA synthesis have pro-cognitive effects in rodents (21, 36, 73, 74).

This study presents some limitations. First, it has been performed only in males. The rationale behind this, was due to the fact that this study aimed at evaluating the possible alterations on KP, following THC exposure during the adolescence and to directly compare the results with the previous findings achieved by prenatally exposing male rats to THC (18). However, considering the well-known sexually dimorphic effects that cannabis consumption during adolescence can induce in cognitive functions, it is possible that adolescent THC exposure differently affects KP metabolism in female rats. This possibility is also corroborated by the fact that sex differences in sleep and arousal have been observed in adult rats born from pregnant dams fed with a diet laced with KYN the last week of gestation (75). Thus, it will be interesting to evaluate, in further studies, the effects of adolescent THC treatment on PFC KYNA levels and cognitive functions in female rats. Second, we tested only one challenge dose of KYN, as well as a single dose of PF-04859989. In addition, the behavioral investigation was performed by using only a single test. This choice is due to the fact that the aim of the behavioral part of the study was to explore, for the first time, the possibility to rescue adolescent THC-induced long-term cognitive impairment with the KAT II inhibitor treatment. Based on the obtained result, it will be interesting to evaluate the effects of different doses of the KAT II inhibitor on THC-induced cognitive impairment by using several behavioral tests, also some relevant to SZ. Finally, in the translational realm, it is relevant to consider that adolescents consuming cannabis are exposed not only to THC, but also to other cannabinoids that could dampen or increase the deleterious consequences of the main psychotropic ingredient of cannabis derivatives.

In summary, the present study revealed that the use of cannabinoids early in life can induce a malfunction of the KP, specifically increased KYNA concentrations, in the adult brain, leading to cognitive impairments in SZ and other major psychiatric disorders. Ongoing experiments are designed to examine this causal connection, and its possible translational significance, in an animal model with abnormal KP metabolism [kynurenine 3-monooxygenase knockout mice; (76, 77)]. Most importantly, the translational relevance of the concept should be carefully assessed in the near future by monitoring the levels of circulating kynurenine, KYNA and other KP metabolites in psychotic patients who consumed marijuana during adolescence.

## Data availability statement

The raw data supporting the conclusions of this article will be made available by the authors, without undue reservation.

## Ethics statement

The study was carried out in accordance with current Italian legislation (D.L. 26/2014) that allows experimentation on laboratory animals only after the approval by Ministry of Health (Rome, Italy), and in strict accordance with the European Council Directives on animal use in research (n. 2010/63/EU).

## Author contributions

SB: conducted research, formal analysis, writing – original draft, writing – review and editing, visualization, conceptualization, and project administration. AI and MZ: conducted research, methodology, formal analysis, writing – original draft, writing – review and editing, and visualization. PD: conceptualization and visualization. LF and RS: conceptualization, visualization, supervision, and funding acquisition. All authors contributed to the article and approved the submitted version.
